# Exome Sequencing Identifies a Novel *MAP3K14* Mutation in Recessive Atypical Combined Immunodeficiency

**DOI:** 10.3389/fimmu.2017.01624

**Published:** 2017-11-27

**Authors:** Nikola Schlechter, Brigitte Glanzmann, Eileen Garner Hoal, Mardelle Schoeman, Britt-Sabina Petersen, Andre Franke, Yu-Lung Lau, Michael Urban, Paul David van Helden, Monika Maria Esser, Marlo Möller, Craig Kinnear

**Affiliations:** ^1^SAMRC Centre for TB Research, DST/NRF Centre of Excellence for Biomedical Tuberculosis Research, Division of Molecular Biology and Human Genetics, Faculty of Medicine and Health Sciences, Stellenbosch University, Cape Town, South Africa; ^2^Division of Molecular Biology and Human Genetics, Faculty of Medicine and Health Sciences, Stellenbosch University, Cape Town, South Africa; ^3^Institute of Clinical Molecular Biology, Kiel University, Kiel, Germany; ^4^Shenzhen PID Laboratory, The University of Hong Kong – Shenzhen Hospital, Shenzhen, China; ^5^Immunology Unit National Health Laboratory Service Tygerberg, Division Medical Microbiology, Department of Pathology, Stellenbosch University, Cape Town, South Africa

**Keywords:** nuclear factor-kappa B-inducing kinase, primary immunodeficiency, tuberculosis, whole exome sequencing, BCG dissemination

## Abstract

Primary immunodeficiency disorders (PIDs) render patients vulnerable to infection with a wide range of microorganisms and thus provide good *in vivo* models for the assessment of immune responses during infectious challenges. Priming of the immune system, especially in infancy, depends on different environmental exposures and medical practices. This may determine the timing and phenotype of clinical appearance of immune deficits as exemplified with early exposure to Bacillus Calmette-Guérin (BCG) vaccination and dissemination in combined immunodeficiencies. Varied phenotype expression poses a challenge to identification of the putative immune deficit. Without the availability of genomic diagnosis and data analysis resources and with limited capacity for functional definition of immune pathways, it is difficult to establish a definitive diagnosis and to decide on appropriate treatment. This study describes the use of exome sequencing to identify a homozygous recessive variant in *MAP3K14*, NIK^Val345Met^, in a patient with combined immunodeficiency, disseminated BCG-osis, and paradoxically elevated lymphocytes. Laboratory testing confirmed hypogammaglobulinemia with normal CD19, but failed to confirm a definitive diagnosis for targeted treatment decisions. NIK^Val345Met^ is predicted to be deleterious and pathogenic by two *in silico* prediction tools and is situated in a gene crucial for effective functioning of the non-canonical nuclear factor-kappa B signaling pathway. Functional analysis of NIK^Val345Met^- versus NIK^WT^-transfected human embryonic kidney-293T cells showed that this mutation significantly affects the kinase activity of NIK leading to decreased levels of phosphorylated IkappaB kinase-alpha (IKKα), the target of NIK. BCG-stimulated RAW264.7 cells transfected with NIK^Val345Met^ also presented with reduced levels of phosphorylated IKKα, significantly increased p100 levels and significantly decreased p52 levels compared to cells transfected with NIK^WT^. Ideally, these experiments would have been conducted in patient-derived immune cells, but we were unable to source these cells from the patient. The functional analysis described in this paper supports previous illustrations of the importance of NIK in human immune responses and demonstrates the involvement of function-altering mutations in *MAP3K14* in PIDs. The genomic approach used for this patient demonstrates its value in the diagnosis of an unusual PID and as a tool for detecting rarer mutations to help guide treatment approaches.

## Introduction

Primary immunodeficiency disorders (PIDs) are heritable genetic errors of the immune system that, if left undiagnosed and untreated, may lead to serious, chronic, and in some cases fatal infections and manifestations of autoimmunity (1, 2). PIDs provide *in vivo* models for identifying factors crucial for human host defense and immune regulation. The nuclear factor-kappa B (NF-κB) family of transcription factors found in mammals is crucial for the expression of developmental, inflammatory, as well as survival genes (3). This pathway consists of a canonical as well as a non-canonical arm, with the former involved in expression of pro-inflammatory genes, and the latter responsible for persistent, slower responses generally not associated with innate immune responses (4, 5).

The non-canonical NF-κB signaling pathway uses NF-κB-inducing kinase (NIK), encoded by mitogen-activated protein kinase (*MAP3K14*), to integrate signals from various membrane receptors, such as tumor necrosis factor alpha receptor family members (6). Several ligands can activate this pathway, including B cell-activating factor, CD40 ligand (CD40L), lymphotoxin beta, receptor activator of NF-κB ligand (RANKL), and TNF-like weak inducer of apoptosis (TWEAK) (7). Binding of these ligands to their appropriate receptors cause NIK to phosphorylate IkappaB kinase-alpha (IKKα), which activates and targets IKKα to p100, its substrate. p100 is in turn phosphorylated by IKKα, which prompts the ubiquitination and partial degradation of p100 to first produce p52 and second permit the formation of RelB-p52 complexes. These heterodimeric complexes move to the nucleus to activate target genes (3). This non-canonical NF-κB pathway controls lymphoid organogenesis, activation of dendritic cells and B cell maturation and survival, and errors in this pathway are associated with lymphoid disorders (5).

Although mortality rates due to *Mycobacterium tuberculosis* infections are increased in mice with genetically disrupted NF-κB, the role NF-κB plays in human immune responses to *M. tuberculosis* is not well understood (8). It has been speculated that some bacterial pathogens misuse specific NF-κB-mediated pathways to promote their survival (9). Activation of autophagy and apoptosis are two processes through which inhibition of NF-κB decreases the amount of intracellular bacilli after *M. tuberculosis* infection (10, 11). Autophagy is associated with innate and adaptive immune responses, as well as inflammation regulation (12). Ineffective autophagy has been implicated in several human diseases, including infectious diseases and inflammatory disorders (13–16). IkappaB kinase (IKK), the regulator of the NF-κB pathway, is required for autophagy activation in mammals, and inhibition of NF-κB increases autophagosome formation (17). Classic NF-κB is not involved in this response, and the mechanism by which IKK promotes stimulus-induced autophagy is largely unknown (18). NIK as well as IKKα are degraded by autophagy when the function of heat shock protein 90, required for the folding and maturation of certain signaling proteins, is inhibited (19). The processing of p100 and NF-κB activity is thus inhibited (20). However, when heat shock stress activates NF-κB, the autophagy pathway is in turn activated, indicating a close interaction and tight regulation between these two pathways (21).

This study identifies a novel potentially disease-causing variant in *NIK* in a South African PID patient using whole exome sequencing (WES). *In silico* analysis predicts the homozygous variant NIK^Val345Met^ to be deleterious and pathogenic. Functional studies with human embryonic kidney (HEK)-293T cells and RAW264.7 cells transfected with either *NIK^WT^* or *NIK^Val345Met^* showed that this mutation significantly affects the kinase activity of NIK, as well as p100 and p52 levels. The relationship between the non-canonical NF-κB signaling pathway and autophagy was also investigated in an attempt to shed some light on their poorly understood interaction. However, *NIK^Val345Met^* did not affect the autophagy pathway.

## Materials and Methods

### Case Report

The proband is a white South African female, from a non-consanguineous marriage, initially diagnosed with humoral immunodeficiency after presenting with a Bacillus Calmette-Guérin (BCG) abscess on the upper leg at the age of 2 years. She received intravenous immunoglobulin (IVIG) replacement therapy. A year later she developed BCG meningitis and received standard treatment in the acute phase with isoniazid (INH), rifampicin (RIF), ethionamide (ETA), and dexamethasone, and thereafter only INH and RIF. At the age of 4 years, diffuse granulomas were identified in her brain. INH, RIF, ETA, and levofloxacin were prescribed for a year, after which the levofloxacin was discontinued. In 2014, at the age of 6 years, she presented with acute loss of consciousness and raised intracranial pressure. *Mycobacterium bovis* BCG genotypically sensitive to INH and RIF was subsequently cultured from the patient. She was started on a very aggressive 18-month treatment regimen of levofloxacin, terizidone, amikacin IV, linezolid, RIF, INH, para-aminosalisylic acid, as well as continued IVIG replacement therapy. With continued dissemination of BCG in spite of the above treatment and in the absence of a confirmed PID diagnostic category, the patient was not selected for bone marrow transplantation. She proceeded to develop severe neurological, motor, as well as cognitive impairment and is now in total dependency care 6 years after initial presentation.

### WES Analysis

The study was approved by the Health Research Ethics Committee of Stellenbosch University (approval no. N13/05/075). Written informed consent was granted by the parents of the patient, and this included genetic evaluation of the patient. The parents also consented to the publication of any molecular findings. The study adhered to the ethical guidelines as set out in the “Declaration of Helsinki, 2013” (22). Venous blood for DNA extraction and WES was drawn from the patient and both of her parents. DNA was purified from blood using the Nucleon BACC3 Kit (Amersham Biosciences, Buckinghamshire, UK).

Enrichment and sequencing of the exomes of the proband and both of her healthy parents were performed with Illumina’s TruSeq Exome Enrichment Kit. It targets >20,000 genes with >200,000 exons as well as 9 Mb of predicted microRNA targets with a total size of 62 Mb. Paired-end WES of the three samples was carried out on the Illumina HiSeq 2000, yielding an average of 120 million reads per sample and resulting in an average coverage of the target regions of 60–80× after duplicate removal. The resulting FASTQ file containing the sequencing data underwent quality control in FastQC and was mapped against the human reference genome hg38 using Burrows-Wheeler Aligner (BWA). PCR duplicates were removed using Picard, while SamTools and Genome Analysis Toolkit (GATK) were used in parallel for the detection of single-nucleotide variants (SNVs). The variant calls from both callers were pooled into a single set, after which ANNOVAR was used for SNV annotation and filtering, and to interrogate a number of programs and databases for each called position to generate more evidence of deleterious mutations. The basic filtering options in ANNOVAR used are (1) filtering out common SNVs unlikely to be disease-causing based on a frequency threshold of >1% in the 1000 Genomes Project (1000GP) data (23) and the Exome Sequencing Project 6500 (ESP6500) data (24); (2) restricting the SNVs to those causing amino acid changes in the protein; (3) assessing the impact on protein structure through prediction tools; and (4) the presence of a gene or SNV in Online Mendelian Inheritance in Man (OMIM) or Human Gene Mutation Database (HGMD), which shows known disease associations. All variants with negative Genomic Evolutionary Rate Profiling (GERP)+++ scores as well as all variants with Functional Analysis through Hidden Markov Models (FATHMM) scores greater than 0.1 were also removed. GERP estimates evolutionary constraints at specific positions in an exome and identifies “constrained elements,” where several positions combine to produce a signal indicative of a putative functional element (25). FATHMM is a high-throughput web-server that can predict the functional consequences of coding and non-coding variants (26). Finally, variants were also removed if they were homozygous in either of the healthy parents of the proband.

### Frequency Investigation

The 1000GP data, ESP6500, and ExAC Browser were investigated for the presence of all potentially disease-causing variants. Only those present in less than 1% of the population in these databases were considered as candidate variants.

### *In Silico* Prediction

Project HOPE [Have (y)Our Protein Explained] (27), PolyPhen-2 (28), Sorting Tolerant From Intolerant (SIFT) (29), and MutationTaster2 (30) were used to predict the functional and structural causes of the amino acid changes on proteins.

### Variant Verification

Sanger sequencing was used to validate the WES results and verify whether the potentially disease-causing variants identified were true variants or sequencing artifacts. Only one potentially disease-causing variant was identified, for which the forward and reverse primers F: 5′AGCCCTGGAAACCTCACC and R: 5′TGAGATTGGCGGAATAAGAGA were used to produce a fragment of 455 bp. This was bi-directionally sequenced at the Central Analytical Facility of Stellenbosch University using the BigDye^®^ Terminator v3.1 Cycle Sequencing kit (Perkin-Elmer, Applied Biosystems Inc., Foster City, CA, USA), followed by electrophoresis on an ABI 3130XL Genetic Analyzer (Perkin-Elmer, Applied Biosystems Inc., Foster City, CA, USA).

### *In Vitro* Functional Analysis

Two plasmids, pWZL-Neo-Myr-Flag-MAP3K14 and pCR-Flag-IKKalpha, were obtained from the non-profit plasmid repository Addgene (https://www.addgene.org/). pWZL-Neo-Myr-Flag-MAP3K14 was a gift from William Hahn & Jean Zhao (Addgene plasmid # 20640) (31) and pCR-Flag-IKKalpha was a gift from Hiroyasu Nakano (Addgene plasmid # 15467) (32). To generate the pWZL-Neo-Myr-Flag-MAP3K14^Val345Met^ mutant construct, the mutation-specific oligo nucleotide primers F: 5′AAGGCAGCGTGAGCTC and R: 5′CAGAGCATGCACTAGGTAT were used together with the Q5^®^ Site Directed Mutagenesis Kit (New England Biolabs Inc., UK) as per the manufacturer’s instructions. Sanger sequencing was used to confirm successful mutagenesis.

### Cell Culture and Transfection

Human embryonic kidney-293T cells and RAW264.7 cells were maintained in Dulbecco’s modified Eagle’s medium (DMEM) containing 4.5 g/L glucose with l-glutamine and supplemented with 10% FBS and 1% penicillin/streptomycin. One day before transfection, the cells were trypsinized and seeded into six-well culture plates so that the cells were 50–80% confluent the following day. Plasmid DNA was purified from the IKKα as well as the wild-type and mutant MAP3K14 plasmids using the PureYield™ Plasmid Miniprep System (Promega Corp., USA) according to the manufacturer’s guidelines. The IKKα plasmid DNA combined with either the wild-type or mutant MAP3K14 plasmid DNA were then transfected into the seeded HEK293T, while only the wild-type or mutant MAP3K14 plasmids were transfected into RAW264.7 cells using Lipofectamine™ LTX Reagent and PLUS™ Reagent (Invitrogen, USA) according to the manufacturer’s instructions.

### BCG Treatment of Cells

The RAW264.7 cells were divided into stimulated and unstimulated groups, each group consisting of untransfected, NIK^Val345Met^-transfected, and NIK^WT^-transfected sub-groups. After transfection of the appropriate plasmids into each sub-group, BCG was added to each well in the six-well plates making up the stimulated group, at a multiplicity of infection of 5. A stock solution BCG with a concentration of 1.46 × 10^6^ CFUs/mL was prepared using BCG Vaccine SSI (Statens Serum Institut, Denmark) and Diluted Sauton SSI (Statens Serum Institut, Denmark). Per well containing 1 × 10^6^ cells growing in 6 mL growing media, 34 µL of this BCG stock solution was added. Cells were stimulated for 16 h at 37°C.

### Bafilomycin Treatment of Cells

The transfected HEK293T cells used for investigation of autophagy were divided into two groups. One received Bafilomycin A1 (Baf) treatment while the other remained untreated. Baf is a known inhibitor of the late phase of autophagy and prevents maturation of autophagic vacuoles by inhibiting fusion between autophagosomes and lysosomes (33). A Baf stock solution with a concentration of 1 mM was made using DMSO and Bafilomycin A1, which was further diluted to 1 µM by adding PBS. Each well in one six-well plate seeded with HEK293T cells received 150 µL of the 1 µM Baf stock and 1,350 µL growth media, and constituted the Baf treatment group. Another plate received only 1,500 µL growth media and constituted the control group. The plates were incubated for 16 h at 37°C.

### Cell Lysis and Western Blotting

All cells were lysed with lysis buffer [0.05 M Hepes, 0.1 M NaCl, 0.01 M EDTA, 0.17 mM Triton X-100, 4 mM Nappi, 2 mM Na_3_VO_4_, and protease inhibitors (Roche)] at 95°C for 5 min and all lysates were stored at −80°C. The lysates were subjected to Sodium dodecyl sulfate polyacrylamide gel electrophoresis SDS/PAGE on Mini-PROTEAN^®^ TGX™ precast polyacrylamide gels [Bio-Rad Laboratories (Pty) Ltd., RSA] containing 1% SDS, after which the proteins were transferred to 0.2-μm-pore polyvinylidine difluoride membranes using the iBlot^®^ Dry Blotting system (Invitrogen, RSA). Membranes were blocked in 5% BSA (w/v) or 5% low fat milk and subsequently probed with the rabbit antibodies phospho-IKKα/β (Ser176/180) (16A6), IKKα, NF-κB p100/p52, NIK (Cell Signaling Technology, Inc., USA; Abcam Inc., UK) and LC3B (Abcam Inc., UK), as well as mouse GAPDH antibody (Santa Cruz Biotechnology Inc., USA). Membranes were then exposed to horseradish peroxidase-conjugated goat anti-rabbit and donkey anti-mouse secondary antibodies (Santa Cruz Biotechnology, Inc., USA) and the proteins were subsequently visualized by enhanced chemiluminescence using Clarity™ Western ECL Substrate (Bio-Rad Laboratories, Inc., USA).

### Statistics

The open platform image analysis tool ImageJ was used for analysis of all western blots. All experiments were done in true biological triplicates and under the same conditions. One-tailed unpaired *t*-tests were used to determine significant differences between samples. All western blots were normalized using GAPDH.

## Results

The patient tested negative for the human immunodeficiency virus as well as Herpes simplex virus and presented with severe hypogammaglobulinemia with decreased immunoglobulin (Ig) levels (IgA < 0.06 G/L, IgM = 0.14 G/L, IgG = 0.42 G/L), increased lymphocyte subset numbers for her age and normal lymphocyte proliferation to mitogens and recall antigens. She was investigated for Mendelian susceptibility to mycobacterial disease (MSMD) by screening *STAT1, LRBA, IL12RB1, IL12B*, and *IFNGR1* for possible disease-causing mutations. No mutations were found in any of these genes. Longitudinal immunological investigation of this patient with persistent, disseminated, treatment-resistant *M. bovis* BCG infection indicated a dramatic decrease of natural killer (NK) cells, B cells, and CD8 cells over time (Table 1). Her CD27^+^IgD^+^ cell population was decreased (2.96%) and class-switched memory B-cells (CD27^+^IgD^−^) were also low (0.12%), while CD40 ligand (CD40L) was detected as present. Memory T cells were low in relation to naïve T cells and reduced levels of γ/δ T cells were observed—cells known to be involved in the innate immune reaction against mycobacteria. The patient’s phytohemagglutinin control lymphocyte proliferation was normal on the T Spot TB test with negative proliferation to specific TB antigens. This is what would be expected, since the test is specific for TB antigens, not BCG—the cause of disease dissemination observed in this patient. Upregulation of CD69 on NK cells after interleukin-2 stimulation was slightly decreased. T cell receptor excision circles (TRECs) and kappa-deleting recombination excision circles were clearly visible (AMPATH Laboratories, South Africa). Investigation by WES was thus pursued to assist with establishing a diagnosis for this patient.

**Table 1:** Normal cell counts versus patient total cell counts at different ages.

Cell counts of the patient at different ages						Reference
Subsets	2 years, 1 month	2 years, 3 months	4 years, 8 months	5 years, 1 months	6 years	2–6 years
Lymphocytes	15,774 (H)	9,522 (H)	6,720 (H)	3,999	2,366	2,340–5,028
T cells	10,719 (H)	6,785 (H)	4,682 (H)	2,655	1,886	1,578–3,707
CD4+	8,743 (H)	5,523 (H)	3,954 (H)	2,258 (H)	1,516	870–2,144
CD8+	1,584 (H)	959	813	533	369 (L)	472–1,107
NK cells	2,549 (H)	1,263 (H)	214	778 (H)	70 (L)	155–565
B cells	2,156 (H)	1,339 (H)	1,783 (H)	398 (L)	362 (L)	434–1,274

*Values are given in cells per microliter*.

*CD, cluster of differentiation; H, high counts; L, low counts; NK, natural killer*.

### Identification of a Homozygous Mutation in NIK

**Figure 1:**
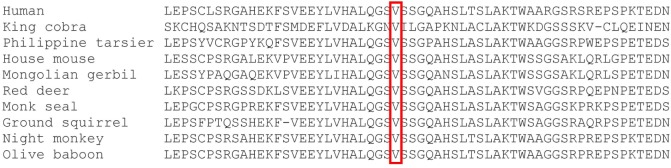
ClustalW multiple sequence alignment. The c.G1033A (p.Val345Met) homozygous mutation identified in this patient is situated at a highly conserved position in *MAP3K14* (34).

Whole exome sequencing of the proband revealed a total of 23,939 variants, which were reduced to 708 candidate variants after filtering and annotation using ANNOVAR (Table 2). Upon exclusion of all variants present in a homozygous state in either of the healthy parents, 9 homozygous and 23 heterozygous variants remained (Table 3). After interrogating OMIM and HGMD, only one variant was identified as potentially disease-causing according to the function of the gene it is situated in. This homozygous c.G1033A p.Val345Met nucleotide variant is situated in exon 5 of *NIK* (Table 4). SIFT and PolyPhen-2 predicted this change to be pathogenic and damaging, while MutationTaster2 was not able to generate any results, indicating the uncharacterized state of *NIK*. The variant is situated at a position that is highly conserved across several different species (Figure 1).

**Table 2:** Variants identified by WES in the proband and both parents.

	Father	Mother	Patient
Total variants	23,440	23,474	23,939
All synonymous and non-frameshifts removed	11,707	11,693	11,925
Remove all variants with a frequency >1% in 1KGP	2,357	2,377	2,495
Remove all variants with a frequency >1% in ESP6500	2,077	2,107	2,160
Remove all variants with negative GERP+++ scores	1,514	1,481	1,535
Remove all variants with positive FATHMM scores	703	688	708
Novel variants	108	113	114
Variants with rs numbers	595	575	594

*ESP6500, Exome Sequencing Project 6500; GERP, Genomic Evolutionary Rate Profiling; FATHMM, Functional Analysis through Hidden Markov Models; WES, whole exome sequencing; 1KGP, 1000 Genome Project*.

**Table 3:** Final list of variants identified in the proband after filtering.

Gene	Variant	ExAC	dbSNP	CADD_phred scores
**Homozygous variants**				
*CELA1*	c.6_7insC; p.V3fs	0.383	–	–
*FOXD4*	c.748_749del:p.G250fs	0.189	–	–
*FOXD4*	c.753_754insCG:p.G252fs	0.189	–	–
*GJD3*	c.C523T:p.H175Y	0.00401	rs202055764	13.57
*GJD3*	c.G758C:p.R253P	0.00286	rs532965992	–
*LRRC46*	c.10_11insGT:p.G4fs	0.000602	rs536101939	–
*MAP3K14*	c.G1033A:p.V345M	–	–	18.77
*NBPF1*	Unknown	0.52	rs2990550	–
*SYN2*	Unknown	0.000729	–	–

**Heterozygous variants**				
*C2CD4C*	c.C205A:p.L69M	0.00134	rs200204713	10.36
*CASP5*	c.67delA:p.R23fs	–	rs372526393	–
*CCDC150*	c.839delA:p.Q280fs	–	rs376590781	–
*CD36*	c.G1016T:p.G339V	0.000602	rs146027667	21.3
*CDC27*	c.C1697T:p.A566V	–	–	36
*CEP164*	c.337delA:p.K113fs	–	–	–
*CES1*	c.A145G:p.I49V	0.303	rs3826193	10.91
*CES1*	c.G53T:p.G18V	0.285	rs3826190	18.35
*CXorf40B*	c.T159G:p.C53W	0.00747	rs140921811	1,305
*FBXW10*	c.T2552C:p.V851A	0.000155	rs199779085	9,638
*FOXD4L1*	c.A463G:p.I155V	0.000705	rs199845792	18.09
*KRT18*	c.C300G:p.S100R	–	–	16.24
*KRT18*	c.C308A:p.T103N	–	–	14.54
*KRT18*	c.C316T:p.R106W	–	rs11551638	14.74
*MTCH2*	c.G196A:p.G66R	–	–	27.9
*OPALIN*	c.G52T:p.A18S	0.00449	rs35821065	25.2
*PAK2*	c.A383G:p.K128R	0.028	rs78043821	21.6
*PLEC*	c.G8992A:p.E2998K	0.000982	rs200898220	7,527
*RASA4, RASA4B*	c.A1054G:p.M352V	0.124	rs144395384	7.05
*SERPINA4*	c.C403T:p.R135C	0.0000244	–	15.31
*SLC11A2*	c.C1291A:p.L431I	0.00251	rs144863268	19.37
*SPDYE6*	c.C890T:p.P297L	0.00348	rs202078839	–
*UBXN11*	c.1104_1181del:p.368_394del	0.301	–	–

**Table 4:** Details of the putative disease-causing variant identified in the proband.

Chromosome	17
Position	45286550
Gene name	*MAP3K14/NIK*
Refseq	NM_003954.4
Reference sequence	G
PROBAND: number of reads with reference	0
FATHER: number of reads with reference	22
MOTHER: number of reads with reference	17
Alternative sequence	A
PROBAND: number of reads with alternative	65
FATHER: number of reads with alternative	26
MOTHER: number of reads with alternative	20
Mutation type	Nonsynonymous SNV
Mutation: DNA (HGVS nomenclature_c.)	c.G1033A
Mutation: protein (HGVS nomenclature_p.)	p.VAL345MET (NP_003945.2)
Prediction < SIFT	Damaging
Prediction < PolyPhen-2	Probably damaging
Prediction < MutationTaster2	N/A
Sanger verification	Yes

*NIK, NF-κB-inducing kinase; SNV, single-nucleotide variant; SIFT, Sorting Tolerant From Intolerant; HGVS, Human Genome Variation Society; N/A, not applicable*.

**Figure 2:**
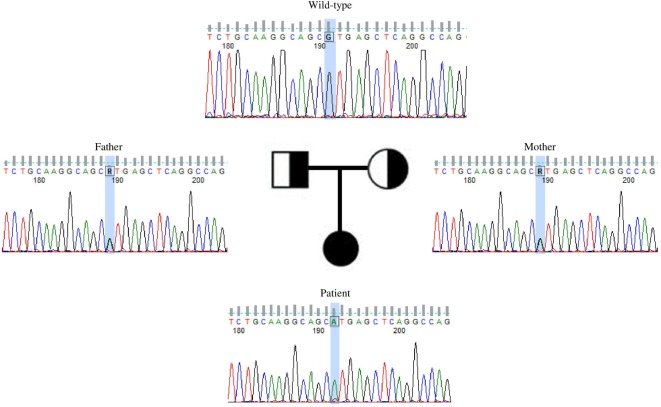
Validation by Sanger sequencing of *NIK^Val345Met^* found by whole exome sequencing. Patient’s c.G1033A homozygous mutation, with one mutated allele inherited from each of the healthy, heterozygous parents.

This variant was covered 65× during WES, improving its likelihood of being a true variant rather than a sequencing artifact. The average coverage for all the variants identified in the proband was 105×. Sanger sequencing confirmed this to be a true variant present in a homozygous state in the patient and heterozygous in both unaffected parents (Figure 2). NIK^Val345Met^ was absent from all interrogated databases and therefore previously unidentified in all investigated populations. No missense or loss-of-function mutations in *NIK* are listed in these databases.

### *In Silico* Variant Prediction

**Figure 3:**
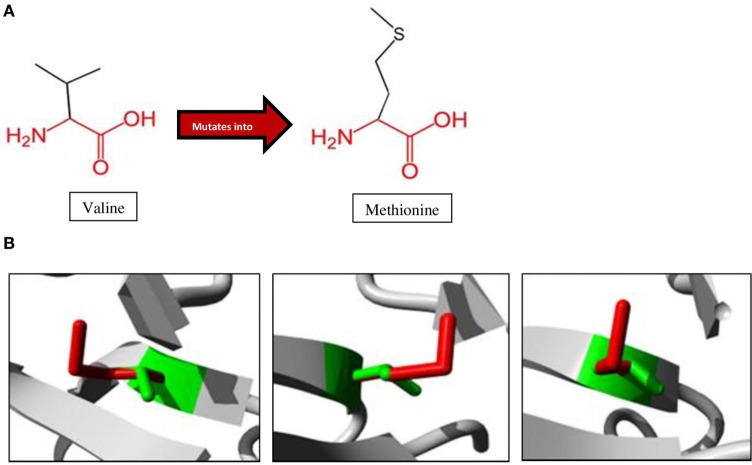
Amino acid changes caused by the mutation identified in the proband. **(A)** Mutation of Val to Met, as observed at position 345 in *NIK* of the patient. The conserved backbone is indicated in red, with the unique side-chain in black. **(B)** Mutant Met (red) is larger than wild-type Val (green), potentially influencing interactions within the protein as well as with other proteins (27).

A schematic representation of the amino acid exchange caused by this variant, with wild-type valine (Val) on the left and mutated methionine (Met) on the right, is shown in Figure 3A. Met and Val are both non-polar, hydrophobic, and aliphatic amino acids. Figure 3B shows the difference in size between Val and Met. The main difference is the presence of a C-beta branch in Val: two non-hydrogen substituents attached to its C-beta carbon, instead of only one as for Met. Met also has an additional sulfur atom, which forms very strong amide N–H⋅S hydrogen bonds crucial for controlling the conformational setting of this amino acid (35). Val is bulkier near the protein backbone and more restricted in the conformations the main-chain can adopt. The Val side chain is extremely non-reactive and is rarely directly involved in protein function, although it can play a role in substrate recognition. The site of variation is situated near a conserved site.

**Figure 4:**
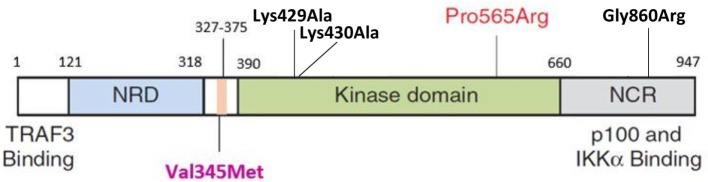
Schematic representation of NIK protein domain structures. The NRD (light blue) contains the BR and the P-RR domains (darker blue). The conserved domain containing V345M (purple label) is indicated in pink and situated before the kinase domain (green). This is followed by the NCR (gray). The P565R amino acid change observed in P1 and P2 by Willmann et al. is situated in the kinase domain. Black labels indicate the mutations previously observed in the murine studies (Lys429Ala; Lys430Ala; Gly860Arg). TRAF3, tumor necrosis factor receptor-associated factor 3; NRD, negative regulatory domain; BR, basic region; P-RR, proline-rich repeat; NCR, non-catalytic region; Lys, lysine; Ala, alanine; Pro, proline; Arg, arginine; Gly, glycine; Val, valine; Met, methionine; IKKα, IkappaB kinase-alpha; NIK, NF-κB inducing kinase (44).

The HOPE analysis indicated that Val345Met is located 45 amino acid positions before the interpro domain known as mitogen-activated protein kinase kinase kinase 14 (IPR017425), associated with protein kinase and transferase activity. This kinase domain stretches from positions 390 to 660 (Figure 4). Because of its close proximity to the kinase domain, it is tempting to speculate that Val345Met may have an effect on the ability of NIK to phosphorylate IKKα. This domain is in contact with residues from other domains, implying the possibility for the variant to influence correct protein function by inhibiting/altering these interactions.

### Plasmid Transfection into Human Cells

To experimentally assess the effect of the mutation of interest, we analyzed the kinase activity of *NIK^Val345Met^* compared to *NIK^WT^* by testing NIK-dependent phosphorylation of IKKα. Sanger sequencing first confirmed that the mutagenesis occurred correctly (Figure S1 in Supplementary Material). IKKα in combination with either wild-type or mutant NIK were transfected into HEK293T cells, while only NIK, in either its wild-type or mutant form, was transfected into RAW264.7 cells. The NIK and IKKα antibodies were then used to show that transfection of the plasmids expressing IKKα and mutant and wild-type NIK into HEK293T and RAW264.7 cells was successful. Untransfected HEK293T cells do not contain endogenous NIK, although they do contain IKKα, while RAW264.7 cells contain both NIK and IKKα endogenously. As seen in Figure S2 in Supplementary Material, there was an increase of both the expression of IKKα in both wild-type and mutant NIK cotransfected HEK 293T cells (although this increase did not reach statistical significance; *p* = 0.0638). Similarly, there was an increase in NIK levels in RAW264.7 cells following transfection with wild-type and mutant NIK constructs (Figure S3 in Supplementary Material). No significant differences are observed between the transfection efficiencies of wild-type- and mutant-transfected cells in either of these cell types.

### Phosphorylation Assay

**Figure 5:**
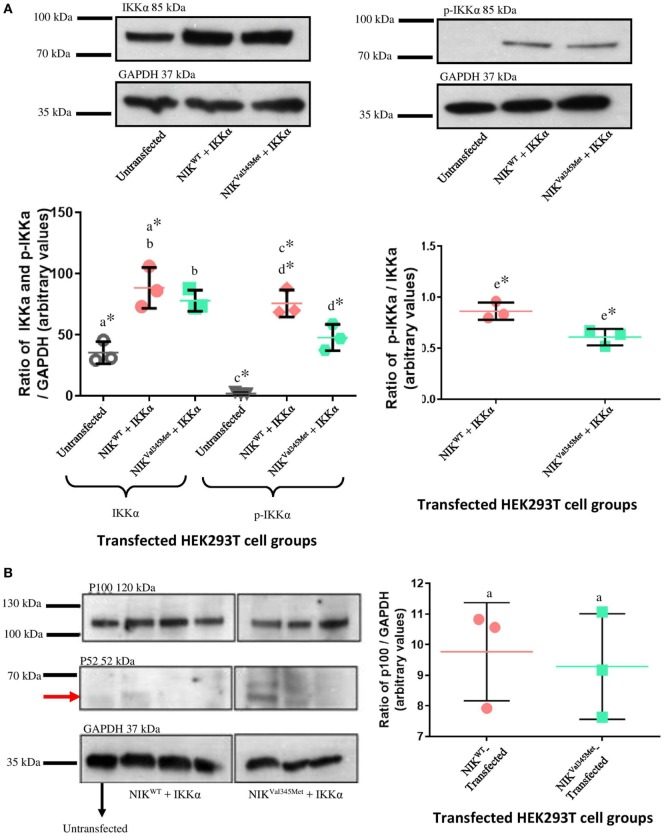

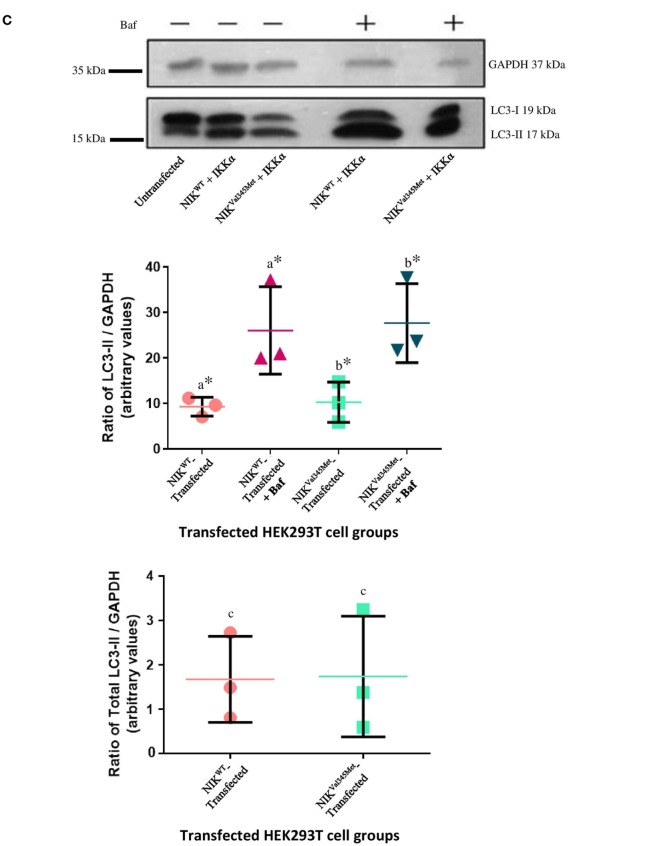
Comparison between NIK^Val345Met^- and NIK^WT^-transfected HEK293T cells. **(A)** No phospho-IKKα was detected in untransfected HEK293T cells, with significant amounts present in transfected cells, proving efficient transfection. A significant decrease in phospho-IKKα is observed in cells transfected with *NIK^Val345Met^* compared to *NIK^WT^*. The ratio of phospho-IKKα over IKKα is also significantly affected by *NIK^Val345Met^*. a: *p* = 0.0084*; b: *p* = 0.3879; c: *p* = 0.0003*; d: *p* = 0.0353*; e: *p* = 0.0199*. **(B)** No quantifiable p52 levels were observed in untransfected, wild-type-transfected, or mutant-transfected samples. p100 was detectable at significant amounts, but no differences between the wild-type and mutant groups were observed. a: *p* = 0.7409. **(C)** Significant differences were seen when comparing cells transfected with wild-type *NIK* and *IKKα* before and after Baf treatment, as well as mutant *NIK* and *IKKα* before and after treatment. However, the difference in autophagy between wild-type-transfected and mutant-transfected groups was not significant. a: *p* = 0.0418*; b: *p* = 0.0366*; c: *p* = 0.9506. Each image is representative of three experiments conducted independently. * indicates significance.

**Figure 6:**
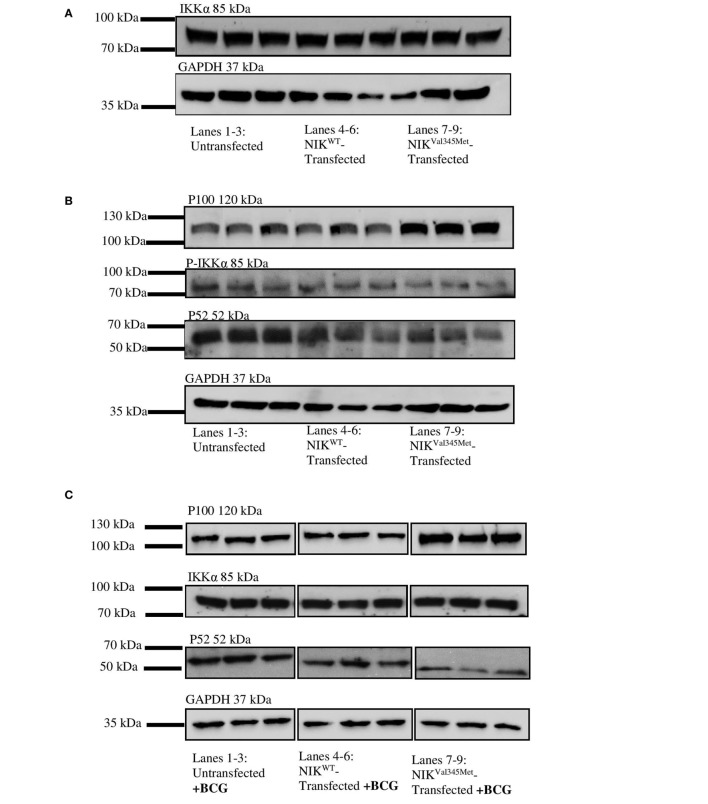

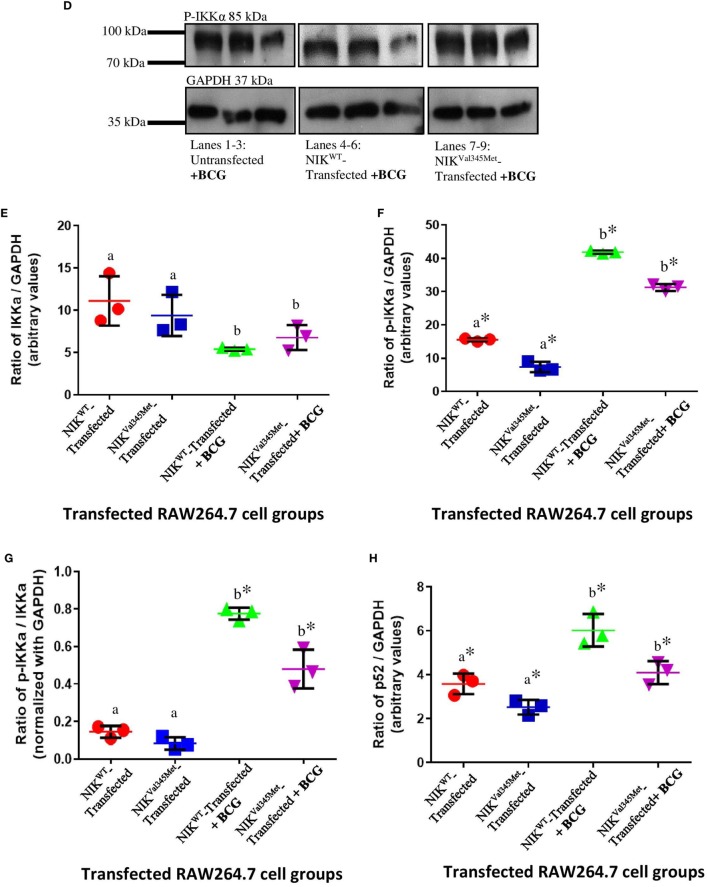

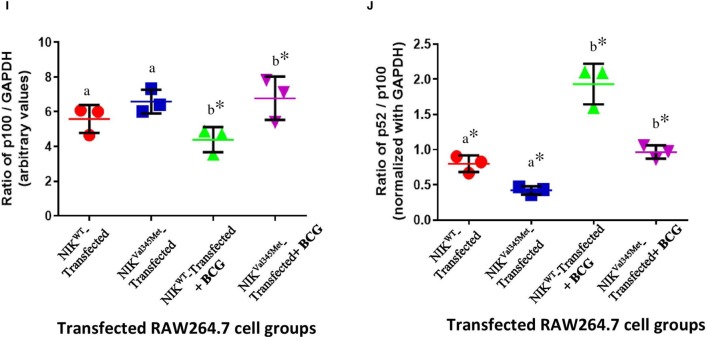
Comparison between NIK^Val345Met^- and NIK^WT^-transfected RAW264.7 cells. **(A,B)** Similar amounts of IKKα was detected in the wild-type and mutant cell groups, indicating that NIK^Val345Met^ had no effect on IKKα production. p100 levels were similar in the wild-type and mutant RAW264.7 cell line groups, with reduced levels of phospho-IKKα and p52 observed in cells transfected with *NIK^Val345Met^* compared to *NIK^WT^*, without BCG stimulation. **(C,D)** p100, p52, and phospho-IKKα levels were affected by the Val345Met mutation, with increased p100, and decreased p52 and phospho-IKKα levels observed in BCG-stimulated cells transfected with *NIK^Val345Met^* compared to *NIK^WT^*. IKKα levels were not affected. **(E)** Quantification of western blots in **(A,C)** showed similar amounts of IKKα in wild-type and mutant cell groups, both without and with BCG stimulation. a: *p* = 0.4757; b: *p* = 0.1844; **(F)** quantification of western blots in **(B,D)** show significant reductions in phospho-IKKα in the mutant-transfected cell groups compared to the wild-type-transfected groups, both without and with BCG stimulation. a: *p* = 0.0010*; b: *p* < 0.0001*. **(G)** The ratio of phosphorylated IKKα over un-phosphorylated IKKα was similar between the wild-type- and mutant-transfected cell groups in the absence of BCG stimulation, but significantly reduced in the mutant-transfected groups compared to the wild-type-transfected groups when stimulated with BCG. a: *p* = 0.0760; b: *p* = 0.0090*. **(H)** Significantly reduced levels of p52 was observed in cells transfected with *NIK^Val345Met^* compared to *NIK^WT^*, both in the absence and in the presence of BCG stimulation. a: *p* = 0.0330*; b: *p* = 0.0208*. **(I)** p100 levels were similar in cells transfected with *NIK^Val345Met^* compared to *NIK^WT^* without BCG stimulation. However, p100 levels significantly increased in the *NIK^Val345Met^*-transfected groups upon BCG stimulation. a: *p* = 0.1745; b: *p* = 0.0458*. **(J)** The ratio of p52 over p100 differed significantly between cells transfected with *NIK^Val345Met^* compared to *NIK^WT^*, both without and with BCG stimulation. a: *p* = 0.0077*; b: *p* = 0.0053*. The data for each image in this figure is representative of three experiments conducted independently. * indicates significance.

IkappaB kinase-alpha and phospho-IKKα/β (Ser176/180) antibodies were used to investigate the difference between IKKα and phospho-IKKα levels in *NIK^WT^* compared to *NIK^Val345Me^*^t^. *NIK^Val345Me^*^t^ was not thought to affect the production of IKKα and as expected, the level of IKKα was not substantially altered by this mutation (Figure 5A: *p* = 0.3879; Figures 6A,C,E; unstimulated: *p* = 0.4757; stimulated: *p* = 0.1844). However, phosphorylated IKKα levels were significantly reduced by *NIK^Val345Met^* in both HEK293T (Figure 5A; *p* = 0.0353) and RAW264.7 (Figures 6B,D,F; unstimulated: *p* = 0.0010; stimulated: *p* < 0.0001) cells, indicating that this mutation alters the kinase activity of NIK. The ratio of phosphorylated IKKα to un-phosphorylated IKKα is shown in Figure 6G, with a significant difference observed between the wild-type-transfected and the mutant-transfected cell groups that have been stimulated with BCG (*p* = 0.0090).

### Downstream Functional Effects of NIK^Val345Met^

Phosphorylated IKKα results in the ubiquitination and proteosomal degradation of p100 to produce p52. The difference in p100 and p52 levels between cells expressing wild-type and mutant NIK was thus also investigated to determine whether *NIK^Val345Met^* has any downstream effects. The ideal would have been to use patient-derived immune cells for these experiments to more accurately measure the downstream effects of the identified mutation. Unfortunately, the patient deteriorated to such an extent that we could not obtain more blood samples from her. HEK293T cells, that are incapable of eliciting an immune response upon infection, were first used. However, no quantifiable levels of p52 were detected (Figure 5B), while no significant differences in the levels of p100 were observed between wild-type- and mutant-transfected groups (Figure 5B; *p* = 0.7409). p100 and p52 levels were also measured in BCG-stimulated and unstimulated RAW264.7 cells transfected with NIK^Val345Met^ and NIK^WT^. In BCG-stimulated cells, p100 levels were significantly increased (*p* = 0.0458) and p52 levels decreased (*p* = 0.0208) in the mutant compared to the wild-type groups (Figures 6C,H,I). The difference in p100 levels between the mutant and wild-type groups was not significant in BCG-unstimulated RAW264.7 cells (Figures 6B,I; *p* = 0.1745), although the decrease in p52 levels remained significant in the unstimulated cells (Figures 6B,H; *p* = 0.0330). The NIK^Val345Met^ variant thus significantly affects the downstream functioning of the non-canonical NF-κB signaling pathway. The ratio of p52 to p100 is shown in Figure 6J, with significant differences observed between the wild-type-transfected and the mutant-transfected cell groups in both BCG-stimulated and unstimulated cell populations (unstimulated: *p* = 0.0077; stimulated: *p* = 0.0053).

### Effect of NIK^Val345Met^ on Autophagy

Autophagy is a dynamic catabolic process during which double-membrane vesicles (autophagosomes) form by engulfing parts of the cytoplasm and subsequently fuse with lysosomes to degrade and recycle their contents (36). LC3 immunoblotting is widely used to measure autophagic activity (37–39). SDS-PAGE and immunoblotting detects two bands of endogenous LC3, namely LC3-I and LC3-II. LC3-I is present in the cytosol, while LC3-II is bound to phosphatidylethanolamine and localized to autophagosomal membranes (40, 41), thus directly correlating with the number of autophagosomes (42). The effect of NIK^Val345Met^ on autophagy was also investigated in this study by measuring LC3-II levels. The western blots and corresponding bar graphs are shown in Figure 5C. Significant differences in LC3-II were observed when comparing cells transfected with NIK^WT^ before and after Baf treatment (*p* = 0.0418), as well as those infected with NIK^Val345Met^ before and after treatment (*p* = 0.0366), thus indicating effective functioning of the autophagic pathway. When comparing the difference in LC3-II levels between mutant and wild-type groups (Figure 5C), however, no significant difference was observed (*p* = 0.9506), indicating that NIK^Val345Met^ has no effect on autophagy.

## Discussion

Phenotype heterogeneity is often observed in patients with mutations in the same gene, and this may in part depend on the patient’s environment (43). We identified a patient with a mutation in *NIK* where the phenotype was modified by early BCG exposure as part of routine vaccination in the first days of life.

Only one previous study to date has described mutations in *NIK* in humans and identified a bi-allelic mutation as the cause of a primary immunodeficiency characterized by multifaceted aberrant lymphoid immunity in two patients (44). Patient 1 (P1) was born to consanguineous healthy parents and had a younger brother who died at the age of 2 years from suspected combined immunodeficiency. Decreased IgG and IgA and elevated IgM levels were identified on a single occasion before any treatment was prescribed. Despite treatment (such as regular intravenous Ig substitution and ganciclovir), the patient continued to suffer from multiple episodes of bacterial and viral infections. There was also one documented episode of granulomatous hepatitis and tuberculosis osteomyelitis due to dissemination after BCG vaccination. An allogeneic hematopoietic stem cell transplantation (HSCT) after reduced toxicity conditioning was performed at the age of 9 years, and upon last report in 2014 she remained clinically well. Patient 2 (P2) is a first-degree cousin of P1 also born to consanguineous healthy parents. She presented with severe chronic diarrhea, recurrent lower respiratory tract infections, as well as oral and esophageal candidiasis. She tested positive for *Cryptosporidium* on one occasion. Her human leukocyte antigen-identical mother was used as a donor for an allogeneic HSCT performed without conditioning at the age of 3 years. As no engraftment was observed after 50 days, the same donor was used and a second transplant was performed. However, the patient died on day 6 following the second HSCT due to rapidly accelerated septic shock and multi-organ failure.

We describe a patient with similar symptoms: initial diagnosis of humoral immunodeficiency with severe hypogammaglobulinemia, decreased memory B cells, B cell lymphopenia, normal T cell proliferation to mitogens and recall antigens, as well as normal IFN-γ production by T cells (Table 1). However, while the patients from the previous study presented with recurrent viral, bacterial, and *Cryptosporidium* infections, the phenotype of our patient seems to be confined to BCG-osis. Our patient did not present with any other infections. WES and subsequent bioinformatics analysis identified a novel putative disease-causing homozygous variant, c.G1033A, situated in *NIK*. *NIK* encodes the 947 residue protein mitogen-activated protein kinase 14, which is a serine/threonine protein kinase involved in NF-κB activity (NM_003954.4). The *NIK^Val345Met^* variant identified in this patient has never been described and is predicted to be disease-causing and deleterious by two *in silico* prediction tools. It is difficult to predict the exact result of this variant on protein function, since *NIK* is poorly characterized. The variant is situated in a highly conserved region (Figure 4). The domain it is located in is important for NIK’s activity and interacts with residues from other domains, making it possible for this mutation to inhibit the correct functioning of NIK. The involvement of *NIK* in B cell development and maturation makes it a perfect candidate gene to investigate further for association with primary immunodeficiency and increased susceptibility to TB.

Phenotypic heterogeneity may be due to different effects of a mutation on protein function (43). However, clinical phenotypic variation cannot always be attributed to different functional consequences of a mutation. As an example, the first patient in whom a mutation in *TYK2* (member of the JAK family of tyrosine kinases) was identified was diagnosed with hyper-IgE syndrome accompanied by lesions of the skin, BCG disease, as well as fungal and viral infections (45). A second patient, also with small deletions in *TYK2* and the absence of protein on western blots like the first patient, presented with BCG disease and brucellosis, but had normal IgE levels and no skin lesions (46). In a further example, three Chinese siblings with intracranial calcifications and epileptic seizures, without severe infectious diseases, were exome sequenced and a mutation in *ISG15* was identified (47). Mutations in this gene were previously found to cause MSMD in three unrelated children from two families in Iran and Turkey (48). Subsequently, intracranial calcifications were also identified in other ISG15-deficient patients. The Chinese individuals never received BCG vaccinations, which could explain why they did not have the MSMD phenotype. Even within a single family, mutations in the same gene have been shown to cause very different clinical phenotypes (35). This is due to the range of infectious or environmental exposures, age of exposure and various modifying epigenetic factors that can affect disease presentation. Therefore, clinical outcomes of patients with immunodeficiencies, even with previously described phenotype/genotype associations, cannot always be accurately predicted.

Phenotypic differences observed between the proband in this study and P1 and P2 described by Willmann and colleagues can also be attributed to the location, and thus the effect, of the identified mutations. The previously identified mutation is situate in the kinase domain of NIK, and completely abolishes the functioning of this protein. The mutation described in this study, however, is situated just before the kinase domain (Figure 4) and does not affect the kinase activity of NIK as critically as the mutation previously described (44). This was proven by the decreased, and not abolished, kinase activity of NIK observed in this study.

The genomic approach in unusual presentations like in the presented patient illustrates its value for successful identification of a novel mutation situated in a PID-causing gene. A confirmed molecular diagnosis directs potential treatment approaches such as the indication for stem cell therapy. Moreover, genetic predisposition combined with protein dysfunction studies are already used to tailor-make patient-specific approaches in mycobacterial disease (49). In the era of personalized medical treatment, based on unique genetic features, it is realistic to anticipate that this will also be extended to the specific treatment of variants of genetically susceptible TB.

## Web Resources

1000GP, http://www.1000genomes.org
ADMIXTURE, http://www.genetics.ucla.edu/software
WANNOVAR, http://wannovar.wglab.org (accessed 3.10.16)
BWA, https://github.com/lh3/bwa
ClustalW, https://www.ebi.ac.uk/Tools/msa/clustalw2/
ESP6500, http://evs.gs.washington.edu/EVS/
ExAC Browser http://exac.broadinstitute.org/
FastQC, http://www.bioinformatics.babraham.ac.uk/projects/fastqc/
GATK suite, www.broadinstitute.org/gatk
HGMD, http://www.hgmd.cf.ac.uk/ac/index.php
MutationTaster-2, http://www.mutationtaster.org/
OMIM, http://www.omim.org/
Picard, http://picard.sourceforge.net
PLINK, http://zzz.bwh.harvard.edu/plink/
PolyPhen-2, http://genetics.bwh.harvard.edu/pph2/
Project HOPE, http://www.cmbi.ru.nl/hope/
SamTools, http://www.htslib.org/
SIFT, http://sift.jcvi.org/

*Accession numbers*: NM_003954.4 and NP_003945.2.

## Ethics Statement

This study was carried out in accordance with the recommendations of the Health Research Ethics Committee of Stellenbosch University (approval no. N13/05/075) with written informed consent from all subjects. All subjects gave written informed consent in accordance with the Declaration of Helsinki. The protocol was approved by the Health Research Ethics Committee of Stellenbosch University.

## Author Contributions

CK, EH, PH, and MM conceived the project. Y-LL carried out all testing on MSMD genes. NS carried out all laboratory work and wrote the first draft of the manuscript. B-SP and AF performed the whole exome sequencing and assisted with the bioinformatics analysis. BG and NS performed the bioinformatics analysis and interpreted the data with CK and MM. MS and MU assisted with the genetic counseling. ME was involved in patient recruitment. All authors read and approved the final manuscript.

## Conflict of Interest Statement

The authors declare that the research was conducted in the absence of any commercial or financial relationships that could be construed as a potential conflict of interest.
